# Molecular Cloning and Biochemical Characterization of a Recombinant Sterol 3-O-Glucosyltransferase from *Gymnema sylvestre* R.Br. Catalyzing Biosynthesis of Steryl Glucosides

**DOI:** 10.1155/2014/934351

**Published:** 2014-08-27

**Authors:** Pragya Tiwari, Rajender Singh Sangwan, Asha, B. N. Mishra, Farzana Sabir, Neelam S. Sangwan

**Affiliations:** ^1^Metabolic and Structural Biology Department, CSIR-Central Institute of Medicinal and Aromatic Plants (CSIR-CIMAP), P.O. CIMAP, Lucknow, Uttar Pradesh 226015, India; ^2^Centre of Innovative and Applied Bioprocessing, (A National Institute under Department of Biotechnology Gov. of India) C-127, Phase-8, Industrial Area, S.A.S. Nagar, Mohali, Punjab 160071, India; ^3^Department of Biotechnology, Uttar Pradesh Technical University, Lucknow, Uttar Pradesh 226021, India

## Abstract

*Gymnema sylvestre* R.Br., a pharmacologically important herb vernacularly called Gur-Mar (sugar eliminator), is widely known for its antidiabetic action. This property of the herb has been attributed to the presence of bioactive triterpene glycosides. Although some information regarding pharmacology and phytochemical profiles of the plant are available, no attempts have been made so far to decipher the biosynthetic pathway and key enzymes involved in biosynthesis of steryl glucosides. The present report deals with the identification and catalytic characterization of a glucosyltransferase, catalyzing biosynthesis of steryl glycosides. The full length cDNA (2572 bp) contained an open reading frame of 2106 nucleotides that encoded a 701 amino acid protein, falling into GT-B subfamily of glycosyltransferases. The GsSGT was expressed in* Escherichia coli* and biochemical characterization of the recombinant enzyme suggested its key role in the biosynthesis of steryl glucosides with catalytic preference for C-3 hydroxyl group of sterols. To our knowledge, this pertains to be the first report on cloning and biochemical characterization of a sterol metabolism gene from* G. sylvestre* R.Br. catalyzing glucosylation of a variety of sterols of biological origin from diverse organisms such as bacteria, fungi, and plants.

## 1. Introduction

**Figure 1:**
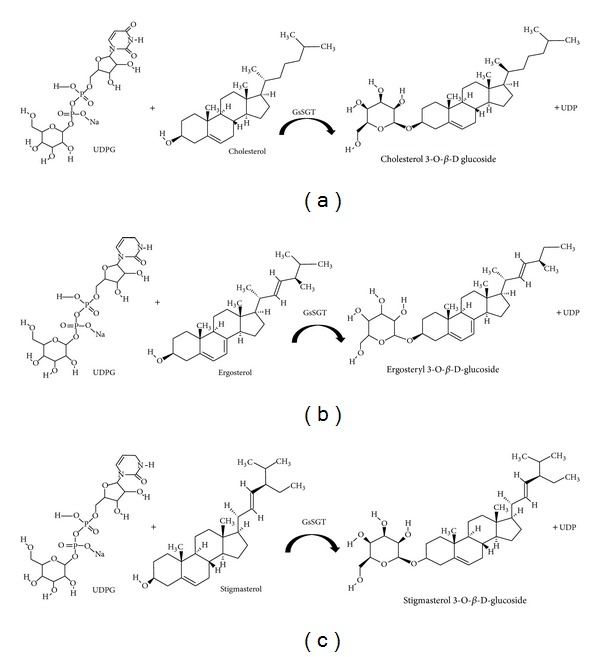
Schematic representation of the glycosylation reaction catalyzed by* G. sylvestre* UDP glucose: sterol glucosyltransferase (GsSGT). UDP-glucose dependent GsSGT catalyzed glucosylation of (a) cholesterol to form cholesterol 3-O-*β*-D-glucoside, (b) ergosterol to form ergosteryl 3-O-*β*-D-glucoside, and (c) stigmasterol to form stigmasterol 3-O-*β*-D-glucoside.

Sterols play significant role in range of biological processes by serving as defense compounds, cell signalling molecules, component of cell membranes, hormones, and precursors of steroid hormones and saponins [1]. Glycosylation of free sterols into their respective glycosides is catalyzed by nucleotide dependent sterol glycosyltransferases or SGTs that are classified as family 1 of the 94 families of glycosyltransferases described in CAZY (carbohydrate active enzymes) database (http://www.cazy.org/) (Figures 1(a), 1(b), and 1(c)). The SGTs in family 1 possess a distinct signature motif at the C-terminal, the PSPG box which represents the binding site for the nucleotide-diphosphate sugar donors while the N-terminal is highly variable, suggesting that the domain might be involved in interaction with diverse sugar acceptors [2]. Family 1 is comprised of GTs implicated in glycosylation of small molecules in plants and is divided into two subsets. The first subset consisting of GTs with PSPG conserved box involved in binding of nucleotide activated sugar donors, consistently present in mammalian GTs which conjugates dietary flavonoids, drugs, steroids, and bile acids [3]. The second subset includes plant GTs involved in sterols and glycerolipids glycosylation [4]. Furthermore, GTs from microbial sources catalyzing biosynthesis of antibiotics such as vancomycin [5], vicenistatin [6], and urdamycin [7] are also classified under family 1 GTs.

Biosynthesis of sterols in plants occurs mainly in the endoplasmic reticulum and recruits mainly mevalonate pathway of isoprenogenesis while some studies also indicate the significant involvement of DOXP or nonmevalonate pathway in sterols and other analogous secondary metabolites such as withanolides or withasteroids [8, 9]. The variation in percentage of sterols in plant cell membranes regulates the homeostasis and thereby maintains optimum fluidity, hydration, and phase behavior [10]. Additionally, properties such as tolerance to freezing, cold acclimation process [11, 12], and stress signaling cascade [13–16] are conferred by the presence of steryl glycosides in plasma membrane. In plants, brassinosteroids function as growth regulators and in development [17] while sterols in* Arabidopsis thaliana* regulate development and gene expression [18]. The sterol biosynthetic pathway intermediates serves as precursor of many secondary metabolites such as triterpene saponins, withanolides and withanosides essentially attributing special physiological traits to the plant like regulating the defense mechanisms against pathogens, herbivory, insect deterrence, and stress-effect combating [8, 9, 13].

Although, a strong literature reporting glycosylation of diverse classes of secondary metabolites is available, there are very few studies on glycosyltransferases catalyzing biosynthesis of steryl glycosides. Several studies have reviewed the biological functions of steryl glycosides, namely, catalytic aspects [19], proteomics [20], structural analysis [21], biochemical studies [1], and structural diversity and occurrence [22]. A comprehensive review on sterol glucosyltransferase [23] provides a detailed study on SGTs, their structural features, and functional relevance particularly in the process of stress responses. Studies at the molecular level on the SGT genes involved in glycosylation of plant sterols have been functionally characterized till date from* Withania somnifera* [24, 25],* Avena sativa*. L [4],* Solanum melongena* [26], and* Arabidopsis thaliana* [27]. Furthermore, three members belonging to the SGT family were identified and their relative expression has been monitored in different organs of* W. somnifera* as well as analyzed as a response to external stimuli [14]. Three SGTs have been identified and isolated by degenerate primers which initiated PCR followed by RACE (rapid amplification of cDNA ends) approach. Elicitor treatments, namely, methyl jasmonate and salicylic acid have been shown to enhance the expression of SGT genes by up to 10-fold suggesting their possible role in defense mechanisms. Furthermore, the expression of SGTs increased on exposure to cold and heat stress highlighting the modification of sterols in abiotic stress mechanisms [14]. Recently, studies on WsSGTL1 from* W. somnifera* demonstrated to enhance salt tolerance, heat tolerance, and cold acclimatization when overexpressed in transgenic* Arabidopsis* plant. The present study highlighted the role of SGTs in plant adaptation to abiotic stress response. Bioinformatics studies further suggested the homology of 3D structure of WsSGTL1 with sterol glycosyltransferase AtSGT of* A. thaliana* [15]. Additionally, recent report showed that overexpression of WsSGTL1 from* W. somnifera* into transgenic tobacco confers biotic and salt stress tolerance and modulates antioxidant system and glycosylation profile in the transgenic plant and it highlights the significance of WsSGTL1 in adaptation of the plant to environmental challenges [16].

Steryl glycosides (SGs), the most abundant glycosylated derivatives of sterols, are ubiquitously present in bacteria, fungi, plants and in some species of animals and include glycosylated triterpenoid, steroidal alkaloids, and steroids [22, 28]. SGs are sugar derivatives of membrane associated sterols and are comprised of a sugar moiety attached to the hydroxyl (−OH) group at the C-3 of a sterol molecule which includes a planar sterol backbone formed by four condensed aliphatic rings and a hydrocarbon side chain at C-17 position [14–16, 20, 26–29]. The studies have suggested that steryl glycosides serve as precursors of defense compounds such as saponins, glycoalkaloids, or avenacosides due to the presence of sugar moieties at various hydroxyl groups [30], C-3 OH position being the most preferred one by SGTs for enzymatic catalysis, followed by 27 *β*-OH position or hydroxyl group present at the side chain of a modified sterol backbone. SGTs have been reported to occur as membrane-associated SGTs [20, 31, 32] by being localized in vacuolar and plasma membranes and as well as cytosolic SGTs. A membrane-bound SGT has been partially purified from* S. melongena *leaves and characterized with respect to its kinetics and molecular properties [26, 33]. The acceptable substrates for the native enzymes included diverse members of C-3 OH steroids, namely, androstane, pregnane and cholestane derivatives, plant sterols, sapogenins, steroidal alkaloids, and triterpene alcohols. Further, a C-3 OH group specific cytosolic SGT has also been isolated from* Panax ginseng* [34] and two SGTs specific for C-3 OH and 27 *β*-OH position have been reported to be purified and biochemically characterized from* W. somnifera* [24, 25, 35].

*G. sylvestre* R.Br. (family: Asclepiadaceae), a widely acclaimed medicinal herb, contains characteristic oleanane and dammarene type triterpene saponins known as gymnemic acids I-XVIII, gymnemasins A-D and gymnemasaponins I-V. The plant parts particularly the leaf tissue and stem are excellent sources of gymnemic acids and the plant extract is used as herbal formulation in regulation of blood sugar levels and hypercholesterolemia. A comprehensive account of the phytochemical and pharmacological properties of* G. sylvestre* R.Br. and its phytoconstituents has been discussed in detail in a recent review [36]. Furthermore, there are several reports on isolation of pure gymnemic acid and analogues and metabolic profiling of the phytomolecules; however literature is void with respect to investigations pertaining to identification, cloning, and catalytic characterization of a gene/enzyme from the plant involved in metabolism including biosynthesis of triterpene glycosides.

Considering the ongoing research on sterol glucosyltransferase and the pharmacological importance of steryl glycosides, the present investigation describes the isolation, heterologous expression in* E. coli* and functional validation of a novel SGT gene (*GsSGT*) from* G. sylvestre* R.Br., wherein the recombinant enzyme has been shown to be capable of catalyzing glucosylationof diverse sterols from bacteria, fungi, and plants. The cloning and characterization of this sterol GT opens up the possibility of glycosylation of important phytosteroid of diverse structural types for their potentially enhanced therapeutic action and improved pharmacokinetics.

## 2. Materials and Methods

### 2.1. Plant Material and Chemicals

*G. sylvestre* R.Br. (gurmar) leaves were collected at different developmental stages from the plants grown in experimental field at CSIR-CIMAP, Lucknow, India. General chemicals and reagents were purchased from Sigma-Aldrich. Reagents and kits of molecular biology were obtained from Invitrogen, Qiagen, Clonetech, Fermentas and Sigma-Aldrich (USA). SYBR green mix was obtained from Applied Biosystems, USA.

### 2.2. RNA Isolation and cDNA Synthesis

Total RNA was isolated from the leaf tissues of the plant by Trizol miniprep method according to manufacturer's protocol. The qualitative and quantitative estimation of the isolated RNA was determined by agarose gel (1.2%) electrophoresis as well as by relative absorbance values at 260 and 280 nm (A_260_/A_280_) in a Nanodrop spectrophotometer. The first strand cDNA was synthesized using Revertaid First strand cDNA synthesis kit (Fermentas) following the manufacturer's guidelines.

### 2.3. Molecular Cloning of* GsSGT*

Multiple sets of degenerate primers were designed from the conserved domains identified in SGT's through multiple sequence alignment of SGT protein sequences retrieved from NCBI and used for isolation of partial* GsSGT* amplicon. The primer combinations used in the study are provided in Table 1. The PCR conditions were as follows: initial denaturation at 94°C for 3 min, followed by 35 cycles at 94°C (1 min), annealing at 55°C (2 min), extension at 72°C (2 min), and final extension at 72°C for 7 min. The RT-PCR products were electrophoresed on 1.2% agarose gel. The resolved fragments were purified through Qiagen columns and cloned in pGEMT vector (Promega) following standard protocols. The putative clones containing amplified fragment were confirmed through sequencing of the plasmid DNA.

### 2.4. Rapid Amplification of cDNA Ends (RACE)

The full length cDNA was obtained by performing 5′ and 3′ RACE-PCR and assembling the respective RACE fragments (Table 1) employing high fidelity Taq polymerase, Fermentas. The PCR program included an initial denaturation of 94°C for 3 min, followed by 35 cycles at 94°C (30 sec), annealing at 55°C (30 sec), extension at 72°C (3 min), and final extension at 72°C for 7 min. The resolved PCR fragment was visualized under ultraviolet light and gel electrophoresed on 1.2% agarose. The PCR amplicon corresponding to putative full length gene was gel purified by Qiagen gel purification kit according to manufacturer's instruction and cloned in pJET vector, Fermentas. The full length clone was confirmed through sequencing of the plasmid DNA.

### 2.5. Bioinformatics Analysis

The evolutionary relationship between GsSGT and reported SGTs was deciphered through BLAST P tool at NCBI database (http://www.ncbi.nlm.nih.gov/). Protein sequence of GsSGT and reported microbial and plant SGTs were subjected to multiple sequence alignment. Various physiochemical properties and protein characteristics were predicted and analyzed (http://www.expasy.org/). A rooted neighbor-joining tree was constructed to determine the phylogenetic relationship using MEGA 5.5 software with 100 bootstrap replicates to establish the confidence level of each node. Conserved domains in the protein were identified by conserved domain database search and Interproscan. Disorderliness of amino acid in protein and the presence of transmembrane segments and secondary structures in the protein were analyzed [37].

### 2.6. Heterologous Expression of the Gene in* E. coli*

The full length cDNA was cloned and expressed in* E. coli* protein expression system, namely, pET-28a as N-terminal fusion protein with a His6 tag to facilitate its purification (Novagen). The complete open reading frame (ORF) was PCR amplified with full length primers (Table 1). The 2.1 kb amplified ORF was cloned at* Nde I* and* Bam H1* restriction sites in pET-28a expression vector and the resulting construct was used to transform* E. coli* BL21 (DE3) strain (Novagen). Transformants were grown at 37°C in LB liquid medium containing 50 mg/mL^−1^ chloramphenicol and 50 mg/mL^−1^ kanamycin as selection antibiotics. The IPTG induced bacterial cells were further grown at 18°C for 12–16 hours (overnight). The induced bacterial culture was centrifuged and the supernatant was discarded. The bacterial pellet was dissolved in protein lysis buffer (containing 1 M potassium phosphate buffer, 10% glycerol, 0.5% Triton X-100, and 10 mM imidazole), vortexed, sonicated and centrifuged at 12,000 rpm for 30 minutes. The his-tagged enzyme in the supernatant was purified by chromatography through a metal-affinity (nickel/NTA) column and the active protein fractions were pooled and analyzed for enzymatic assays.

### 2.7. Sterol Glucosyltransferase Assay

Biochemical characterization of partially purified GsSGT protein was performed through enzymatic assays by glucosylation of sterols into their respective steryl glucosides employing unlabelled uridine diphospho glucose as nucleotide sugar donor. The standard reaction mixture containing 20 mM Tris, pH, 6.5, 10 *μ*M UDP-glucose, 8.5 *μ*M sterol, and 0.7 *μ*g of protein was taken for the GsSGT enzymatic assay. The reaction was initiated by addition of the enzyme and the reaction mixture was incubated at 37°C for 1 hour. At the end of the reaction, the reaction contents were extracted with 2 mL of ethyl acetate and evaporated to dryness at room temperature. The samples were redissolved in methanol, spotted on a TLC plate, and run using chloroform: methanol (85 : 15 mL) as the mobile phase. The plates were developed with anisaldehyde spray reagent and chromogenically visualized after heating at 110° with the respective sterol and glucoside standards cochromatographed on the same TLC. Control reactions (heat inactivated enzyme and substrate control) were set up in a similar manner to identify and ascertain the product formation to be through the enzymatic reaction catalyzed by the SGT (GsSGT). The catalytic parameters for GsSGT protein were optimized for temperature, pH, linearity with time, protein concentration, substrate saturation, and enzyme's thermostability. For quantitative estimations of catalytic activities the HPLC based analysis were performed and product formed was computed as the appearance of peaks corresponding the product in the optimized condition using modified protocol (21).

### 2.8. Tissue Specific Expression Analysis

The expression profiles of* GsSGT* were examined in the tissues at different developmental stages through real-time PCR studies with normalization of values against *β*-actin gene as control. The reaction was set up in total volume of 20 *μ*L containing template of* GsSGT* and actin. PCR program included following thermal cycles: 40 cycles of 95°C for 10 sec, 95°C for 15 sec, and 55°C for 1 min while the reaction conditions for melting curve were comprised of 95°C for 15 sec, 50°C for 1 min, and 95°C for 15 sec, respectively. Further, the specificity of the reaction was determined through dissociation curve analysis compared with no-template controls (the reactions were run in triplicates) with two biological repeats in StepOne RealTime PCR system (Applied Biosystems) using SYBR green mix (Applied Biosystems). Subsequently, the relative expression was represented by the relative quantification value, calculated by 2^−ΔΔ*C*^ *t* method (22).

## 3. Results and Discussion 

### 3.1. Cloning of* GsSGT* Gene from* G. sylvestre*

The partial* GsSGT* cDNA (370 bp) was isolated from a cDNA library of* G. sylvestre* leaves employing a homology based PCR screening approach. Further, 3′RACE resulted in a 685 bp partial clone showing homology with WsSGTL1 from* W. somnifera*. The partial clones were submitted to Genbank at NCBI database (Genbank ID: GU181368; GU191124). 5′RACE and 3′RACE partial fragments (1.7 kb and 968 bp, resp.) and the internal fragment sequences were collated to yield an assembled full length* GsSGT*consisting of 2572 nucleotides including 2106 open reading frame (ORF) and 5′ and 3′ untranslated regions (UTRs) of 228 bp and 199 bp, respectively. The 2106 bp ORF corresponded to a polypeptide of 701 amino acids. BLAST P (http://blast.ncbi.nlm.nih.gov/Blast.cgi) similarity search revealed predominant homology of* GsSGT* with SGTs from* W. somnifera* WsSGTL1 (69%),* A. thaliana* 3-*β*-SGT (70%),* M. truncatula* 3-*β*-SGT (70%),* Z. mays* 3-*β*-SGT (68%), and* A. sativa* UDP-glucose: sterol glucosyltransferase (57%), suggesting its close sequence resemblance with members of the family 1 glycosyltransferases.

### 3.2. Phylogenetic Studies

**Figure 2:**
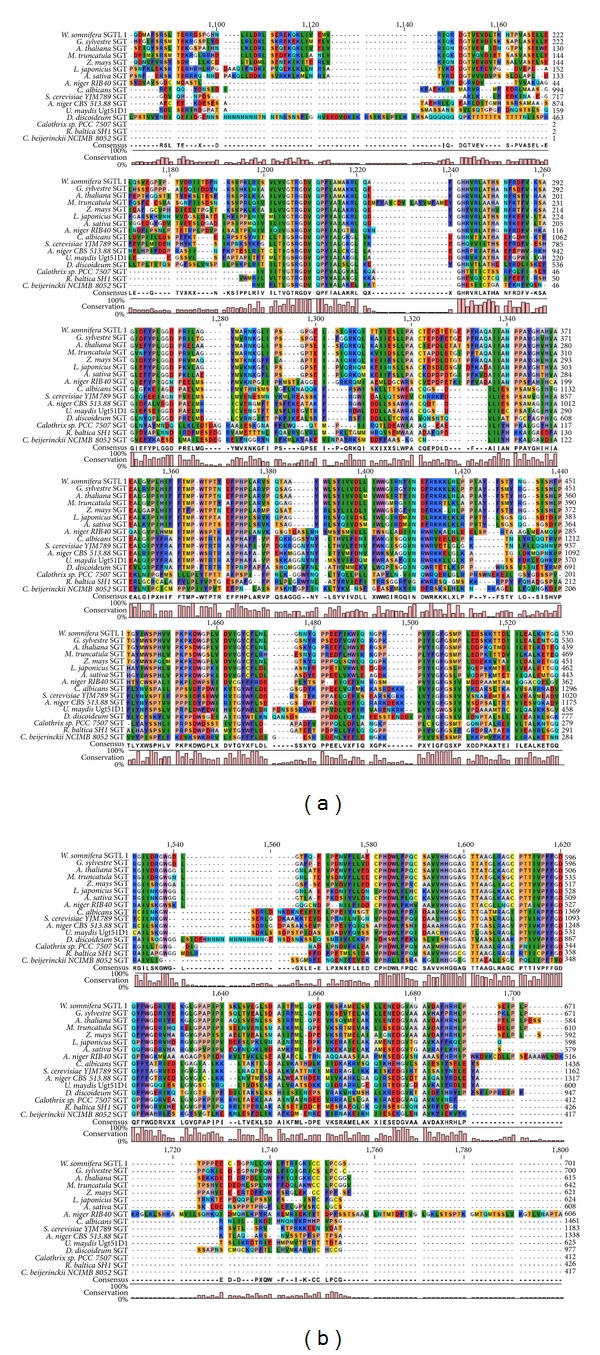
Multiple sequence alignment of protein sequences of microbial and plant SGTs and* GsSGT*. The SGT accessions used for the alignment are as follows: WsSGTL1 [*W. somnifera*]: accession number ABC96116.1, 3-*β*-SGT [*A. thaliana*]: accession number AEE31976.1, 3-*β*-SGT [*M. truncatula*]: accession number XP003592094.1, 3-*β*-SGT [*Z. mays*]: accession number ACG25324.1, sterol glucosyltransferase 1 [*L. japonicas*]: accession number AEX55299.1, UDP-glucose: sterol glucosyltransferase [*A. sativa*]: accession number CAB06081.1, sterol glucosyltransferase [*A. oryzae RIB40*]: accession number XP001818661.2, UDP-glucose: sterol glucosyltransferase [*C. albicans*]: accession number AAD29571.1, UDP-glucose: sterol glucosyltransferase [*S. cerevisiae YJM789*]: accession number EDN59412.1, 3-*β*-SGT [*A. niger CBS 513.88*]: accession number XP001391739.2, UDP-glucose: sterol glucosyltransferase Ugt51D1 [*U. maydis*]: accession number AAN77910.1, sterol glucosyltransferase [*D. discoideum*]: accession number AAD28546.1, 3-*β*-SGT [*C. sp. PCC 7507*]: accession number AFY35770.1, UDP-glucose: sterol glucosyltransferase [*R. baltica SH 1*]: accession number NP869730.1, and 3-*β*-SGT [*C. beijerinckii NCIMB 8052*]: accession number ABR34421.1. The consensus is represented by conserved amino acid residues and percentage of conservation by a bar graph, respectively. The alignment at the N and C terminal ends showed negligible conservation and is therefore not included in the figure.

Multiple sequence alignment was performed at the protein level with sterol glucosylating GTs from microbes and plant, which revealed large variations in the N-terminal regions suggesting that plant SGTs have diverged during evolution from microbial SGTs functionally involved in sterol glycosylation and evolved to accommodate diverse acceptors (Figure 2). The C-terminal region of SGTs remains mostly conserved with some variations, while the conservation pattern highlights conservation of different amino acid residues in microbial and plant SGTs (Figure 2). This inference is in accordance with the similar catalytic mechanism and region-selectivity for 3-OH group of sterols, experimentally validated from SGT enzyme assays performed for GsSGT and WsSGTL1 [35]. This is in agreement with the fact that the C-terminal domain of GTs represents the nucleotide activated sugar donor binding site and remained structurally conserved during the course of evolution, UDP glucose (sugar donor) being the most preferred by SGTs in plants, followed by UDP-galactose, UDP-rhamnose, UDP-xylose, and UDP-glucuronic acid, respectively. Structural investigations assigned GT-B family to the GsSGT with structure consisting of two separate Rossmann domains connected through a linker region which represents the catalytic site of the enzyme. It is interesting to note that the GT-B family had shown remarkable structural conservation in the C-terminal sugar donor binding domain while variations were found in the N-terminal regions as well as in the helices, loops, and active site which have evolved naturally to accept diverse molecules [38]. This might be a possible explanation for broad functionality of some GTs while elucidating regiospecificity of others [38]. Additionally, presence of transmembrane helices was detected in the secondary structure of GsSGT protein which constitutes about 28.48% of the structure (Supplementary Figure 1; Supplementary Material is available online at http://dx.doi.org/10.1155/2014/934351).

**Figure 3:**
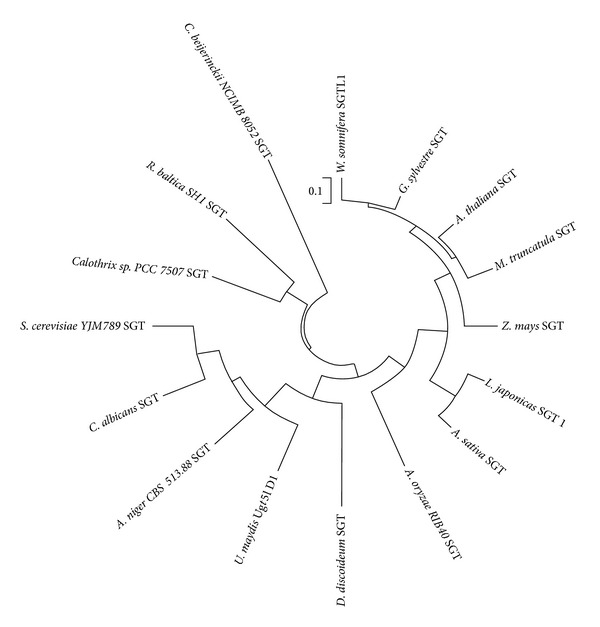
Phylogenetic analysis of GsSGT with Neighbour-Joining method using MEGA 5.5 software. The protein sequences retrieved from NCBI database included in the analysis comprised plant and microbial SGTs: WsSGTL1 [*W. somnifera*]: accession number ABC96116.1, 3-*β*-SGT [*A. thaliana*]: accession number AEE31976.1, 3-*β*-SGT [*Medicago truncatula*]: accession number XP003592094.1, 3-*β*-SGT [*Z. mays*]: accession number ACG25324.1, sterol glucosyltransferase 1 [*L. japonicas*]: accession number AEX55299.1, UDP-glucose: sterol glucosyltransferase [*A. sativa*]: accession number CAB06081.1, sterol glucosyltransferase [*A. oryzae RIB40*]: accession number XP001818661.2, UDP-glucose: sterol glucosyltransferase [*C. albicans*]: accession number AAD29571.1, UDP-glucose: sterol glucosyltransferase [*S. cerevisiae YJM789*]: accession number EDN59412.1, 3-*β*-SGT [*A. niger CBS 513.88*]: accession number XP001391739.2, UDP-glucose: sterol glucosyltransferase Ugt51D1 [*U. maydis*]: accession number AAN77910.1, sterol glucosyltransferase [*D. discoideum*]: accession number AAD28546.1, 3-*β*-SGT [*C. sp. PCC 7507*]: accession number AFY35770.1, UDP-glucose: sterol glucosyltransferase [*R. baltica SH 1*]: accession number NP869730.1 and 3-*β*-SGT [*C. beijerinckii NCIMB 8052*]: accession number ABR34421.1.

A neighbor-joining phylogenetic tree was generated to establish the phylogenetic relationship of GsSGT with reported microbial and plant SGT protein sequences through MEGA 5.5 software (Figure 3) [39]. The study demonstrated that microbial SGTs, namely, 3-*β*-SGT from* Clostridium beijerinckii* NCIMB 8052: accession number ABR34421.1. was the progenitor during evolutionary divergence of SGTs involved in sterol glucosylation. Further, SGTs from* Rhodopirellula baltica SH1* accession number NP869730.1 and* Calothrix sp. PCC7507* accession number AFY35770.1 were the successors which diverged from* Clostridium* SGT. The evolutionary trends suggested that plant SGTs are the recent members of SGT family and originated from fungal SGTs [39].

Phylogenetic studies have also revealed some interesting findings on the glucosyltransferase from* G. sylvestre*. The GsSGT protein was found to be evolutionarily related to the antibiotic GTs from microbes (Gtfd). Since the SGTs and antibiotic GTs are both classified in family I GT, therefore it is hypothesized that GsSGT from the plant could have pharmacological prospects, playing a role in biosynthesis of steryl glucosides as well as precursors of triterpene saponins. Considering the pharmacological significance of steryl glycosides in plants and their biosynthesis being catalyzed by UDP dependent sterol glucosyltransferases, cloning of* GsSGT* and its novel biochemical characteristics are of key significance. This investigation constitutes the first progress on understanding steryl glucoside biosynthesis in* G. sylvestre* R.Br. Also,* G. sylvestre* 3-O-sterol glucosyltransferase was found to be the youngest member in phylogenetic evolution and grouped in the same clade as the* W. somnifera* WsSGTL1 (Figure 3). The similar properties of both proteins indicated that these are closely linked with a parallel evolution and it was further evident from their catalytic/functional similarities, both exhibiting a preference for C-3 hydroxyl (−OH) sterols as the glucosyl moiety acceptor substrates.

### 3.3. Heterologous Expression

**Figure 4:**
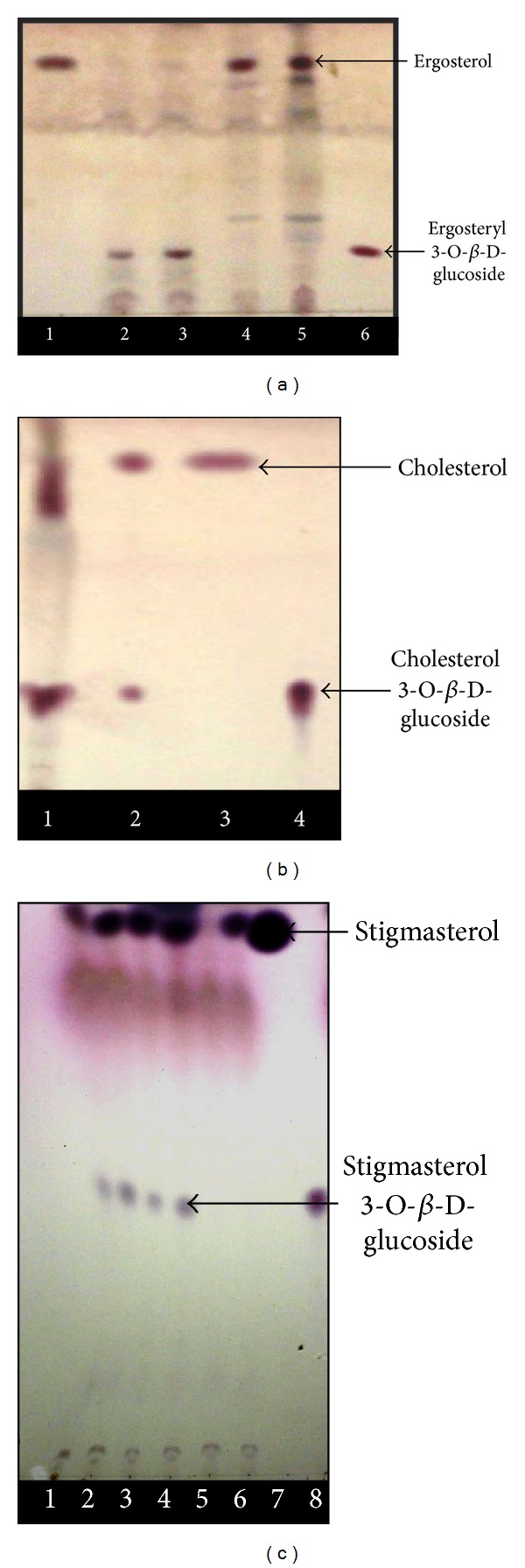
Thin layer chromatographic identification of different steryl glycosides (cholesterol 3-O-*β*-D-glucoside, ergosterol 3-O-*β*-D-glucoside, and stigmasterol 3-O-*β*-D-glucoside) formed in the GsSGT catalyzed reaction with UDPG as glucosyl donor and respective sterols as acceptor substrates. (a) Lane 1, ergosterol standard; lane 2 and lane 3, enzyme assay performed with purified GsSGT protein; lane 4, heat inactivated enzyme control; lane 5, substrate (negative) control; lane 6, ergosterol 3-O-*β*-D-glucoside standard. (b) Lane 1, enzyme assay performed with crude enzyme extract; lane 2, enzyme assay performed with purified GsSGT protein; lane 3, cholesterol standard; lane 4, cholesterol 3-O-*β*-D-glucoside product standard. (c) Lane 1 and lane 2, GsSGT enzymatic assay (Tris buffer, pH 6.5); lane 3 and lane 4, GsSGT enzymatic assay (Tris buffer, pH 7.5); lane 5, control (Tris buffer + enzyme); lane 6, control 2 (Tris buffer + stigmasterol + protein, no UDP-glucose); lane 7, stigmasterol standard; lane 8, cholesterol 3-O-*β*-D-glucoside standard.

**Figure 5:**
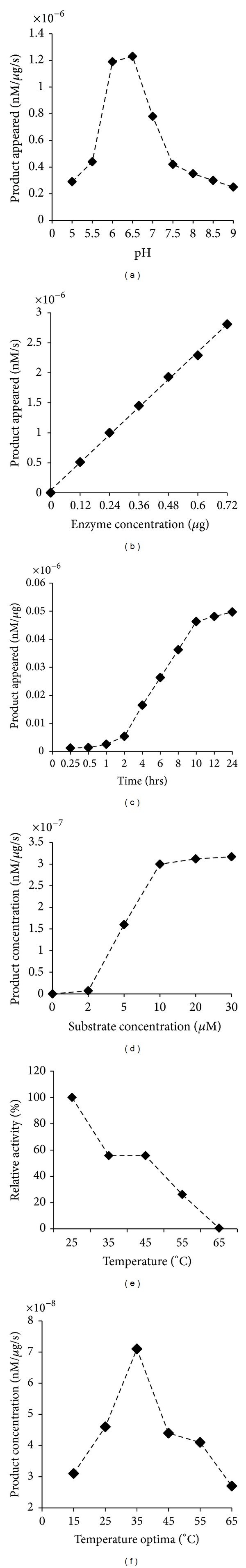
(a) Enzyme preparation protein versus rate of reaction product formation. (b) Time of incubation versus amount of product formed. A time course experiment was performed at various intervals of time, namely, 0 hours, 15 minutes, 30 minutes, 1 hours, 2 hours, 4 hours, 6 hours, 8 hours, 10 hours, 12 hours, and 24 hours with 37°C as the temperature of incubation. (c) Assay pH versus catalytic activity of GsSGT. (d) substrate saturation curve of GsSGT for aglycon (ergosterol). (e) Thermostability of the recombinant GsSGT. (f) Optimum temperature for enzymatic actvity of GsSGT.

The complete open reading frame of* GsSGT* was PCR amplified, subjected to double-digestion with* Nde 1* and* Bam H1* restriction enzymes and heterologously expressed in pET-28a with a T7 promoter and a His-tag for protein purification. The induced protein showed overexpression at 18°C with IPTG. The nickel/NTA affinity purified protein encoded a recombinant protein of 77 kDa and was catalytically characterized through enzymatic assays employing UDP-glucose as sugar donor and diverse sterols, namely, cholesterol, ergosterol, and stigmasterol as substrates in the enzymatic assays (Figures 1, 4, and 5).

### 3.4. Biochemical Characterization 

#### 3.4.1. Thin Layer Chromatography (TLC) Based Identification of Catalytic Reaction Product

The optimized TLC protocol was used for identification of steryl glucosides, the products of the enzyme catalyzed reaction. TLC analysis with authentic standards indicated the formation of cholesterol 3-O-*β*-D-glucoside, ergosteryl 3-O-*β*-D-glucoside, and stigmasterol 3-O-*β*-D-glucoside which comigrated with the standard. The results showed the enzymatic preference of GsSGT for hydroxyl (−OH) group at C-3 of sterols which is consistent with the previous reports on SGTs [35]. Also, it is interesting to note that* G. sylvestre* UDP: sterol glucosyltransferase exhibited significant activity with cholesterol as acceptor substrate. This is in comparison with the catalytic traits of* W. somnifera*, WsSGTL1 which exhibited no activity with cholesterol. The inference can be drawn on the fact that variations in amino acid residues at the N terminal substrate binding site might be responsible for enzyme's catalytic preference for cholesterol. Significant activity was also seen with ergosterol and stigmasterol leading to their glucoside formation by glucosyl conjugation at their C-3 OH position. This suggests that GsSGTcatalyzes O-glucosylation at C-3 hydroxyl position of sterols resulting in formation of ergosteryl 3-O-*β*-D-glucoside and stigmasterol 3-O-*β*-D-glucoside. TLC based chromogenic identification of the glucoside products with authentic standards and controls showed the formation of glucoside product which was absent in control (Figures 4(a), 4(b), and 4(c)). The steryl glucoside formation was further validated by HPLC analysis. Various catalytic parameters of the enzyme were determined and validated through enzyme assays with the respective controls (Figures 5(a), 5(b), 5(c), 5(d), 5(e), and 5(f)) leading to the optimization and identification of the glucosylated sterols.

## 4. Tissue Specific Expression Analysis

**Figure 6:**
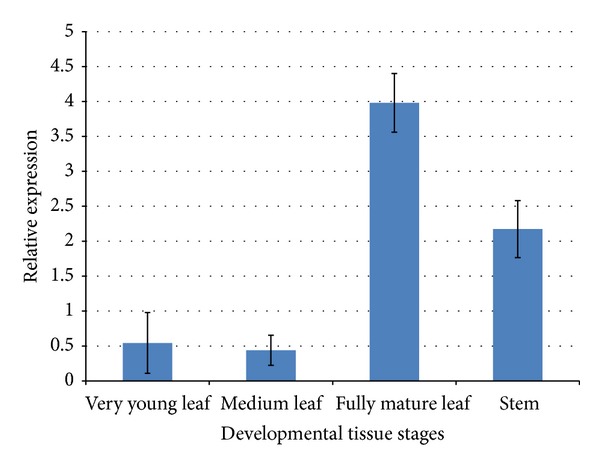
Real-time PCR based analysis of expression profile of* GsSGT* gene in* G. sylvestre* stem and leaf at different developmental stages using normalization with *β*-actin gene as internal control. The values are the mean ± standard deviation from three independent replications of the experiment.

The expression profile of the sterol glucosyltransferase (*GsSGT*) was examined in different tissues of* G. sylvestre* and the expression was found to be consistent in all the developmental stages, namely, very young leaf, mature leaf, fully mature leaf, and stem tissues with a low expression in fully mature leaf. Further, real-time PCR of* GsSGT* expression in different developmental stages revealed that* GsSGT* transcript levels were relatively highest in fully mature leaf followed by stem tissue and very young leaf (Figure 6).

Studies have reported the presence of a highly conserved PSPG domain at the C-terminal (44 amino acid residues), involved in binding of nucleotide activated sugar donor (UDP-glucose in this study). Also, the presence of HCGWNS motif within the PSPG box is essential for enzymatic activity [40] and the identification of the respective motif in GsSGT protein validated the findings. The presence of HCGWNS as a signature motif was found to be 100% conserved in GsSGT protein sequence, consistent with other SGTs classified in family 1 glycosyltransferases (Supplementary Figure 2). Further, studies have suggested that the sugar donor and acceptors are accommodated in the cavity between the N and C terminal domain and the formation of substrate binding pocket is due to several regions in the primary sequence of the protein [41]. Additionally,* in silico* motif diversity analysis in all the GT sequences and phylogenetic evolution of the PSPG domain was earlier reported [42]. This was further ascribed to the presence of high percent of hydrophobic amino acid in the secondary structure of GsSGT (alanine—6%, isoleucine—7%, leucine—9%, proline—8%, and valine—6%) which suggest that the aliphatic side chain plays an important role in enzyme catalysis and function, forming the hydrophobic core of the protein (Supplementary Figure 3). Furthermore, the hydrophobic interactions play an important role in protein folding. During polypeptide folding, the nonpolar amino acids come together in close proximity and excluded from polar environment. The water molecules are released from interior leading to increase in entropy which contributes to protein folding.

The phylogenetic position analysis suggested the cytosolic localization of GsSGT as transmembrane domain was not present and its phylogenetic position was far away from the membrane-associated SGTs. Such proteins have polar group on their surface which interact with water molecules while the hydrophobic or nonpolar amino acid residues are buried in the interior and constitutes the active site of the enzyme. Protein secondary structure was predicted through psipred server and consisted of 28.48% alpha helix, 20.00% extended strands, and 51.52% random coil, the remaining secondary structures being negligible (Supplementary Figure 4). The presence of alpha helix provides stability to the secondary structure of the protein. Also, an alpha helix represents hydrogen bond formed between an amino group and 3rd amino group of same polypeptide chain and forms a stable and ordered structure in protein. Furthermore, amino acid residues such as lysine, alanine, methionine, leucine, and glutamate tend to adopt a helical conformation in the protein. The extended strand or antiparallel *β*-sheet consists of hydrogen bonds formed between peptides bonds in antiparallel polypeptide chain. Glycine and proline residues have the propensity to form *β*-sheets in a protein. It connects more regular secondary structures like alpha helix in a protein. The presence of a random coil is undefined and indicates an absence of regular secondary structure like alpha helix or beta sheet. Random coils do not constitute the catalytic core of the protein and found outside the active site, not involved in catalytic mechanisms (Supplementary Figure 4).

Sterol metabolism accounts for a primary process essential in all organisms including bacterial and fungal species. Steryl glycosides (SG) have been reported from several bacterial species such as* H. pylori* [43],* A. axanthum* [44],* S. citri* [45], and* B. hermsi* [46]. Studies also suggested the role of SGs in pathogenesis in bacteria [47]. A cholesterol alpha-glycosyltransferase, cloned from* H. pylori* causes infections of gastric pathology such as carcinoma and peptic ulcers. The glycosylation of the cholesterol by SGTs results in immune system invasion by the pathogen [48]. The mutants with deleted copy of the gene showed absence of cholesterol glycosylation and impaired pathogenesis. Regarding fungal SGTs; these enzymes have been reported to be involved in selective degradation of peroxisomes (pexophagy) in* P. pastoris* while UGT51 enzyme in* Y. lypolytica*, is required for utilization of decane. Defect in decane assimilation occurs due to defective copy of UGT51 sterol glucosyltransferase. These studies have suggested that sterol metabolism constitutes a primary metabolic process in microbial species. However, in plants, glycosylation of sterols into the respective steryl glycosides comprises of secondary metabolic pathway activity as well, among others [49]. Sterol glucosyltransferases or SGTs constitutes key regulators of sterol glucosylation mechanism and their presence is often species specific. It has been suggested that the presence of steryl glycosides in plasma membrane imparts properties such as freeze tolerance, the cold acclimation process, and stress signaling cascade [11–16]. It was hypothesized from the deduced phylogenetics that evolution favored the secondary compartmentalization of sterol metabolism giving preference to more significant primary metabolic processes essential for cell-survival. Further, plant SGTs evolved from microbial SGTs exhibiting better adaptability and evolutionary selection in comparison to microbial SGTs.

Sterols and their glycosylated derivatives, SGs, constitute an integral component of plant secondary metabolism network. The presence of steryl glycosides confers various properties to the plant, essential for adaptation and cell survival. Sterol glycosyltransferases or SGTs are the less explored members of GT superfamily and very few reports are available on research pertaining to SGTs. However, literature highlighting the wider relevance of SGs ranging from pharmacological properties, namely, anticancer activity to apoptotic, cytotoxic, antistress and antimicrobial activity, studies on biochemical and structural investigations involving SGTs would be a prime area in drug development utilizing plant formulations. Further, such studies exploring both the structural aspects of the protein and biochemical characterization would help in establishing the functional role of an enzyme in biosynthesis of phytomolecules in plants. Also, the presence of bioactive secondary metabolites and elucidation of the biosynthetic role in synthesis of glycol-conjugates would enrich the studies of plant GTs in glycoengineering.

Additionally, the cloning and characterization of 3-OH sterol GT opens up the possibility of glycosylation of important sterol structure based phytomoleclues of diverse structure for potential therapeutic functions.

## 5. Conclusions

Sterol glycosyltransferases are important class of enzymes, catalyzing sterol glucosylation and steryl glucosides formation* in planta*. The addition of a carbohydrate moiety to secondary metabolites enhances several biological properties making glycoconjugates as key drugs or drug-derivatives in pharmacological studies. The present study discusses the cloning and biochemical characterization of a UDP dependent glucosyltransferase catalyzing the biosynthesis of steryl glucosides in* G. sylvestre* R.Br., an Ayurvedic herb with antidiabetic properties. The complete ORF was expressed in pET-28a and the gene was found to encode a polypeptide of 77 kDa as observed with SDS-PAGE. It matched well with the computed molecular mass of the protein. Further, the protein exhibited catalytic preference for C-3 position OH groups of sterols leading to formation of cholesterol 3-O-*β*-D-glucoside and ergosteryl 3-O-*β*-D-glucoside. There are recent observations suggesting the occurrence of ergosterol and derivatives in medicinal plant,* W. somnifera* [50] and its glycosylation by SGTs [35]. Although, till now, ergosterol was regarded as a fungal metabolite, literature highlighting the presence of ergosterol and derivatives and their isolation from plants are being increasingly explored and reported [50]. Furthermore, glucosylation of stigmasterol (phyto-constituent present in* G. sylvestre* R.Br.) to form stigmasterol 3-O-*β*-D-glucoside by GsSGT suggest its role in biosynthesis of these glycoconjugates of these phytosteroids. Real-time PCR studies demonstrated that the expression of* GsSGT* was consistent in all developmental stages of tissues suggesting that biosynthesis of steryl glucosides and presence of sterols as precursors of triterpene saponins constitute an essential metabolic network in plant secondary metabolomics in* G. sylvestre* R.Br. This SGT from* G. sylvestre* R.Br. is unique as it possesses the capacity to glucosylate different kinds of sterols present in lower organisms like bacteria and fungi to higher organisms (plants and animals). This study highlights significant possibilities focusing on sterol metabolism and its evolution from microbes to higher organisms and would further aim at pathway engineering of sterol metabolism. This research finding reports for the first time identification, isolation, and characterization of a novel SGT from* G. sylvestre*, capable of glucosylating all major sterols expectedly playing diverse role in primary as well as secondary metabolism.

## Supplementary Material

Supplementary Figure 1: Deduced disorderliness state of the protein, secondary structures and presence of transmembrance helices in the GsSGT protein.
Supplementary Figure 2: Analysis of the nucleotide (UDPG) binding PSPG conserved domain in *G. sylvestre* UDP: sterol glucosyltransferase. The HCGWNS motif within the PSPG box is essential for enzymatic activity and found to be 100% conserved in the protein sequence of GsSGT.
Supplementary Figure 3: Pie chart representation of the percentage of each amino acid present in secondary structure of GsSGT protein.
Supplementary Figure 4: Protein secondary structure analysis showing 28.48% alpha helix, 20.00% extended strands and 51.52% random coil.

## Figures and Tables

**Table 1:** Details of primers used for PCR amplification of *GsSGT* ORF, heterologous expression, and real-time expression profiling.

Primer details	Primer's name	Nucleotide sequence of primer (5′-3′)	Length (mer)	Tm (°C)	GC content (%)	Size of the amplicon
Primers used for partial clone, full length cDNA and heterologous expression						
Degenerate	WsSGTF2	TGGCTACATCTGGACTCCTCAT	22	57.3	50.0	370 bp
	WsSGTR2	TGGTTCATCACGGTGGTGCT	20	59.4	55.0	
3′RACE	GymGTF1	GGCACTGGAGAATACAGGACAGAG	24	58.8	54.2	684 bp
	GymGTF2	CCTCGTTGATTGCCCTCATGACTG	24	59.9	54.2	
5′RACE	GymGTR1	GGTTTAGGCCCCTTCTGTATCCAC	24	59.2	54.2	1.7 kb
	GymGTR2	CCAACAACATCAACCAGAGGGCC	23	60.6	56.5	
	GymURD_0_1	AGAGACTCGATGATGGCCTTGAGC	24	60.5	54.2	
	GymURD_0_2	AATCACTGAAGTTAGCATGAGTAGCCAGCC	30	61.6	46.7	
	GymURD_0_3	ACTCCTGTAATTTCTTAGCCATAGCC	26	56.5	42.3	
Complete ORF	2.3GymFLF1	TTGGATTAACATATTTGAGCATCCAGGC	28	57.6	39.3	2.1 kb
	2.3GymFLF2	TTGAGCATCCAGGCTTCTCTTGCATGG	27	62.8	51.9	
	2.3GymFLR1	ATTCTGTAATTCCTTGTTCCGTTGTAAACC	30	57.5	36.7	
pET-28a expression	PTGGTF_0_N	**CATATG**GATTCTGATGGGACTGGC	24	57.4	50.0	2.1 kb
	PTGGTR_0_B	**GGATCC**CTAAGAACAACAAGGCAGG	25	59.4	52.0	

Primers used for semiquantitative and real-time PCR expression studies						
RT-PCR	Actin3F	TTGCCGAGATAATGGCCTAC	20	54.6	50.0	250 bp
	Actin3R	TACCGCGACTTCGATCTTTT	20	54.0	45.0	
	SGTF2	TGGCTACATCTGGACTCCTCAT	22	57.3	50.0	370 bp
	SGTR2	TGGTTCATCACGGTGGTGCT	20	59.4	55.0	
Real-time PCR	PTGymGTF2	CCTCGTTGATTGCCCTCATGACTG	24	59.9	54.2	350 bp
	PT1.8GymFL1	GGATCCCTAAGAACAACAAGGCAGGCTG	28	62.7	53.6	

∗Restriction sites for pET-28a expression are highlighted in bold and underlined.

## Abbreviations

GsSGT: * Gymnema sylvestre* 3-O-sterol glucosyltransferase;
CAZY: Carbohydrate active enzyme database;
UDP-GTs: Uridine diphosphoglucose dependent glucosyltransferases;
PSPG: Plant secondary product glucosyltransferase;
PCR: Polymerase chain reaction;
LB: Luria broth;
IPTG: Isopropyl *β*-D-thiogalactopyranoside;
SDS-PAGE: Sodium dodecyl sulphate-polyacrylamide gel electrophoresis;
HPLC: High performance liquid chromatography;
3D: Three-dimensional;
CDD search: Conserved domain database search.
