# Supplementary material for: Molecular Cloning and Biochemical Characterization of a Recombinant Sterol 3-O-Glucosyltransferase from Gymnema sylvestre R.Br. Catalyzing Biosynthesis of Steryl Glucosides
Source: Biomed Res Int. 2014 Aug 27;2014:934351. doi: 10.1155/2014/934351 (PMC4163426; doi:10.1155/2014/934351)
Supplement: Supplementary file 1 — Supplementary Figure 1: Deduced disorderliness state of the protein, secondary structures and presence of transmembrance helices in the GsSGT protein. Supplementary Figure 2: Analysis of the nucleotide (UDPG) binding PSPG conserved domain in G. sylvestre UDP: sterol glucosyltransferase. The HCGWNS motif within the PSPG box is essential for enzymatic activity and found to be 100% conserved in the protein sequence of GsSGT. Supplementary Figure 3: Pie chart representation of the percentage of each amino acid present in secondary structure of GsSGT protein. Supplementary Figure 4: Protein secondary structure analysis showing 28.48% alpha helix, 20.00% extended strands and 51.52% random coil. [file 934351.f1.pdf]

Supplementary Figures

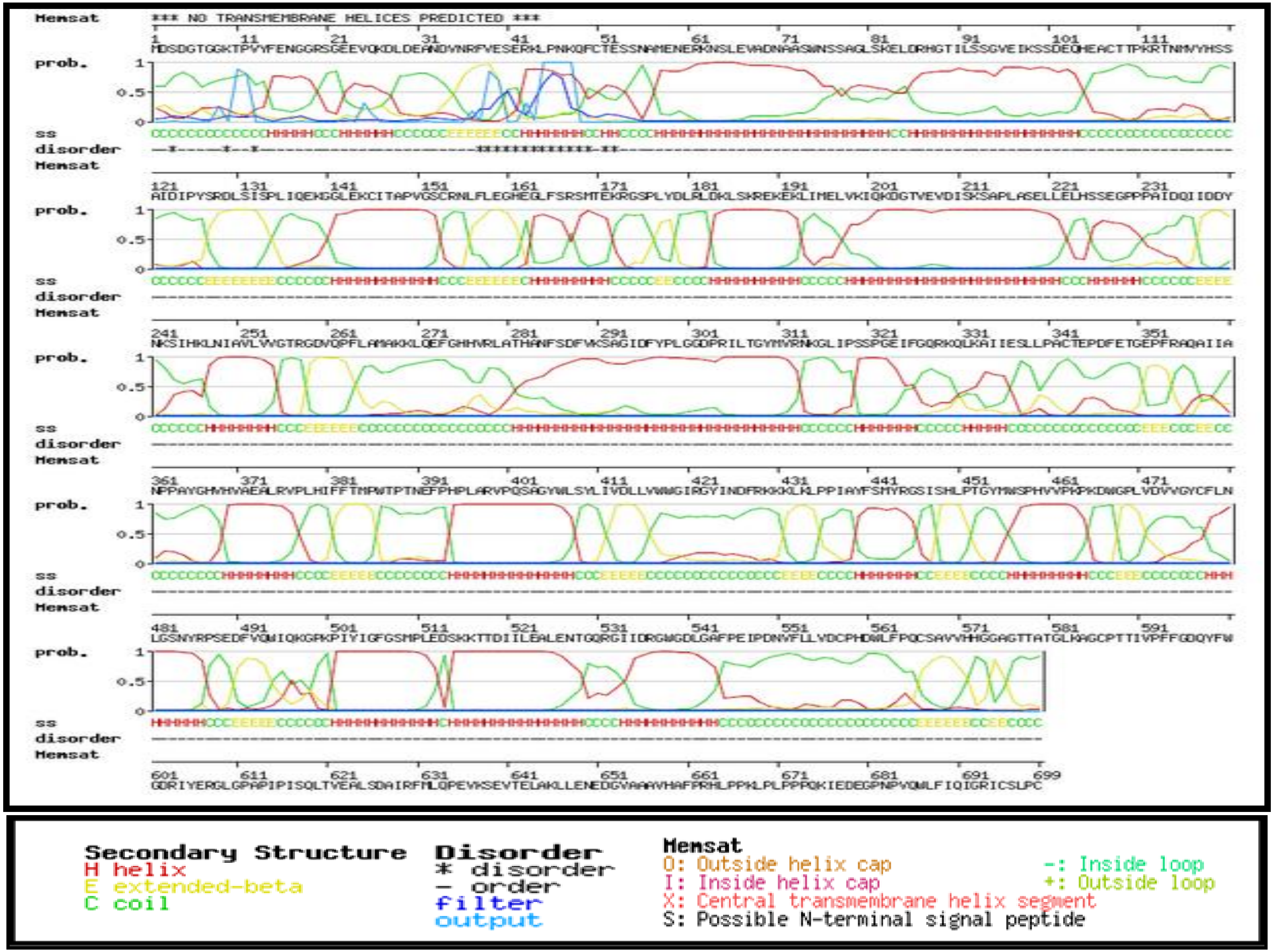

SUPPLEMENTARY FIGURE 1:

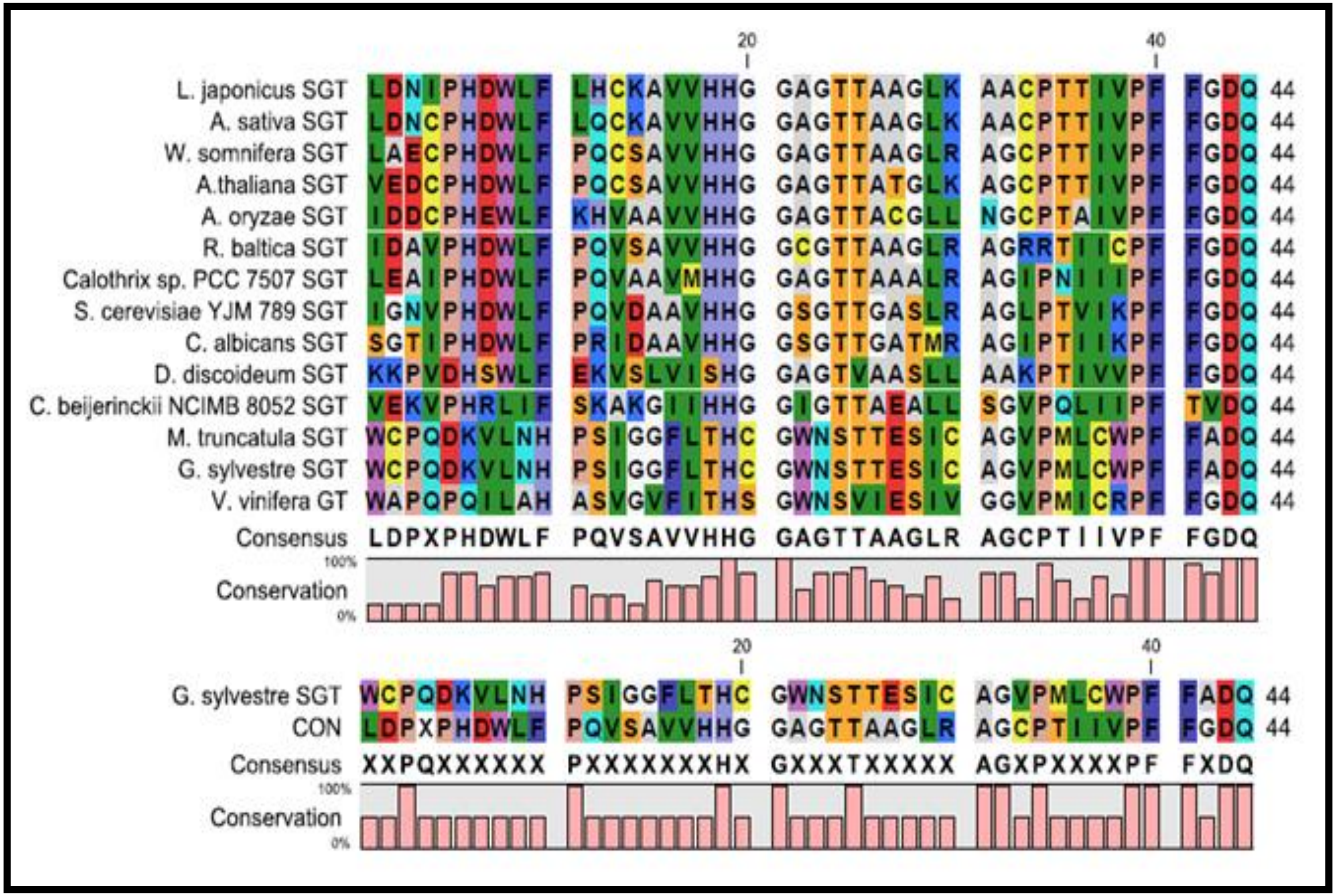

SUPPLEMENTARY FIGURE 2:

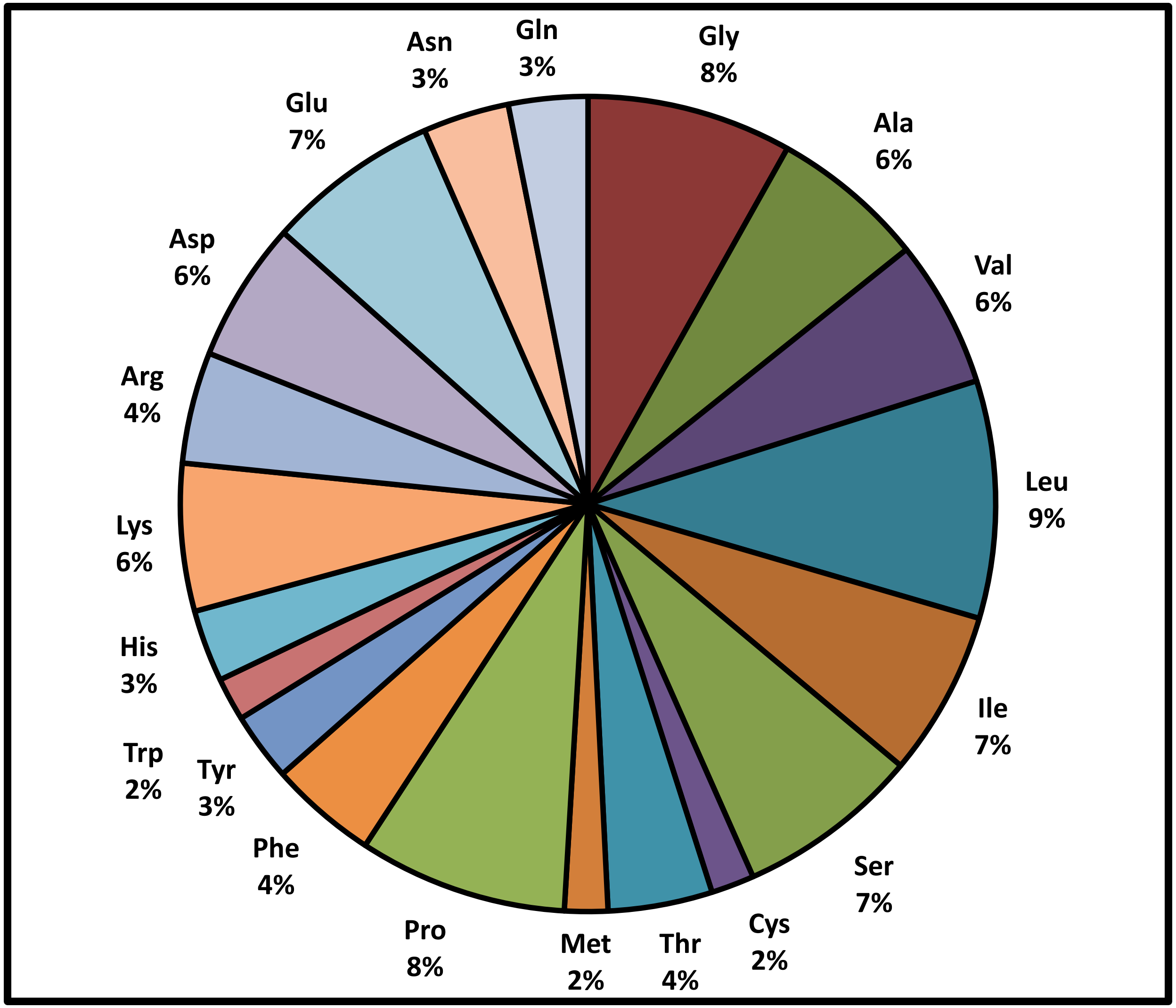

SUPPLEMENTARY FIGURE 3

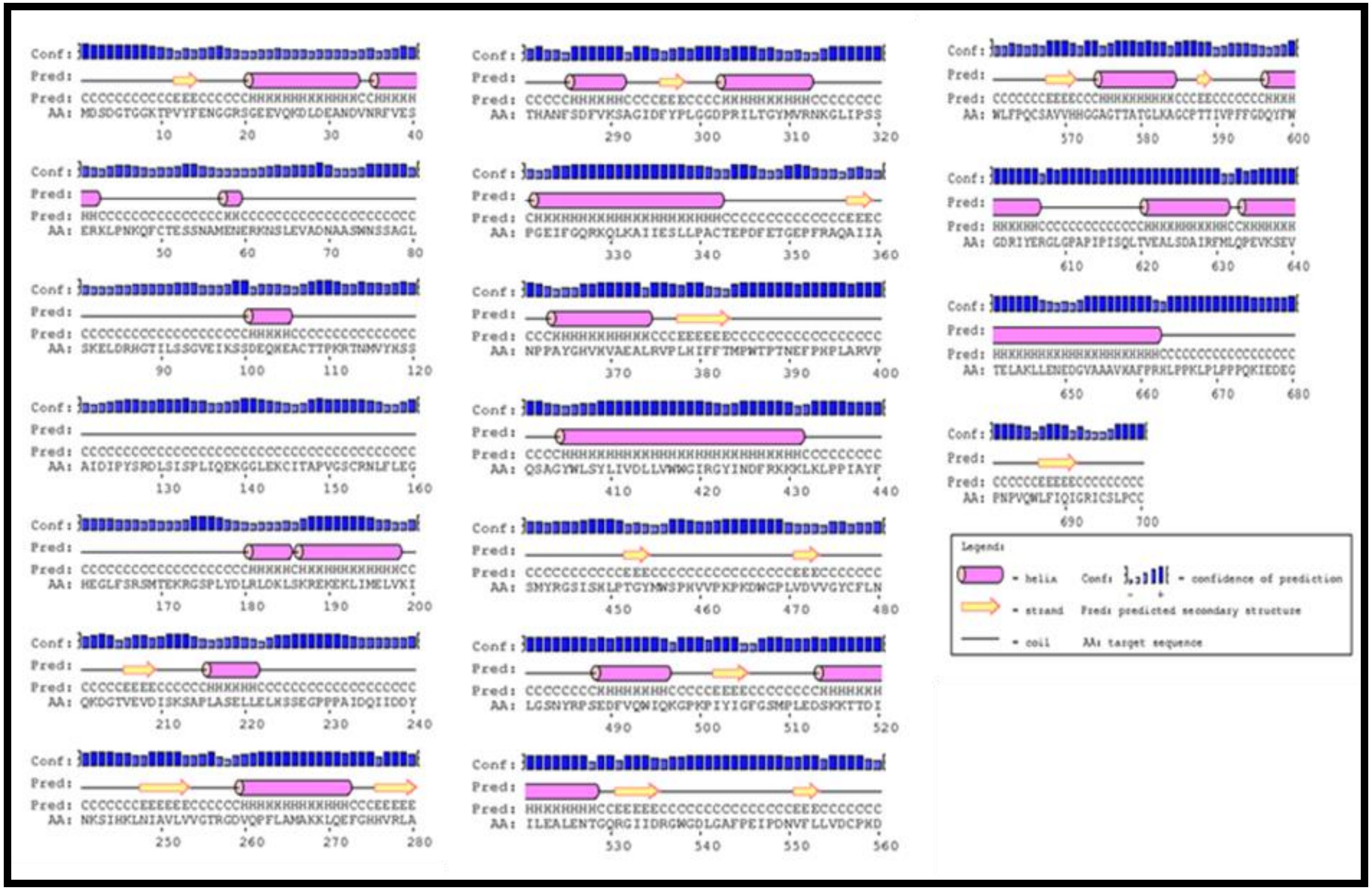

Secondary structure in GsSGT	Percentage of occurrence
Alpha helix	29.53
Extended strand	19.54
Random coil	50.93

SUPPLEMENTARY FIGURE 4:
